# Supplementary material for: Generalizable compound protein interaction prediction with a model incorporating protein structure aware and compound property aware language model representations
Source: Commun Chem. 2025 Dec 19;9:37. doi: 10.1038/s42004-025-01844-0 (PMC12823658; doi:10.1038/s42004-025-01844-0)
Supplement: Supplementary file 2 — Supplementary Information [file 42004_2025_1844_MOESM2_ESM.pdf]

Supplementary Information
Generalizable Compound Protein Interaction Prediction with a Model Incorporating Protein Structure Aware and Compound Property Aware Language Model Representations

Yiming Zhang[1], Mizuki Takemoto[2], Ryuichiro Ishitani[3], and Atsuhiro Tomita[2,*]

[1] Department of Information and Communications Engineering, School of Engineering, Institute of Science Tokyo, Yokohama, Kanagawa 226-8503, Japan

[2] Preferred Networks, Inc., Chiyoda-ku, Tokyo, Japan

[3] Department of Computational Drug Discovery and Design, Medical Research Institute, Tokyo Medical and Dental University, Bunkyo-ku, Tokyo, Japan, Department of Biological Sciences, Graduate School of Science, The University of Tokyo, Bunkyo-ku, Tokyo, Japan, Preferred Networks, Inc., Chiyoda-ku, Tokyo, Japan

*To whom correspondence should be addressed: atomita@preferred.jp.

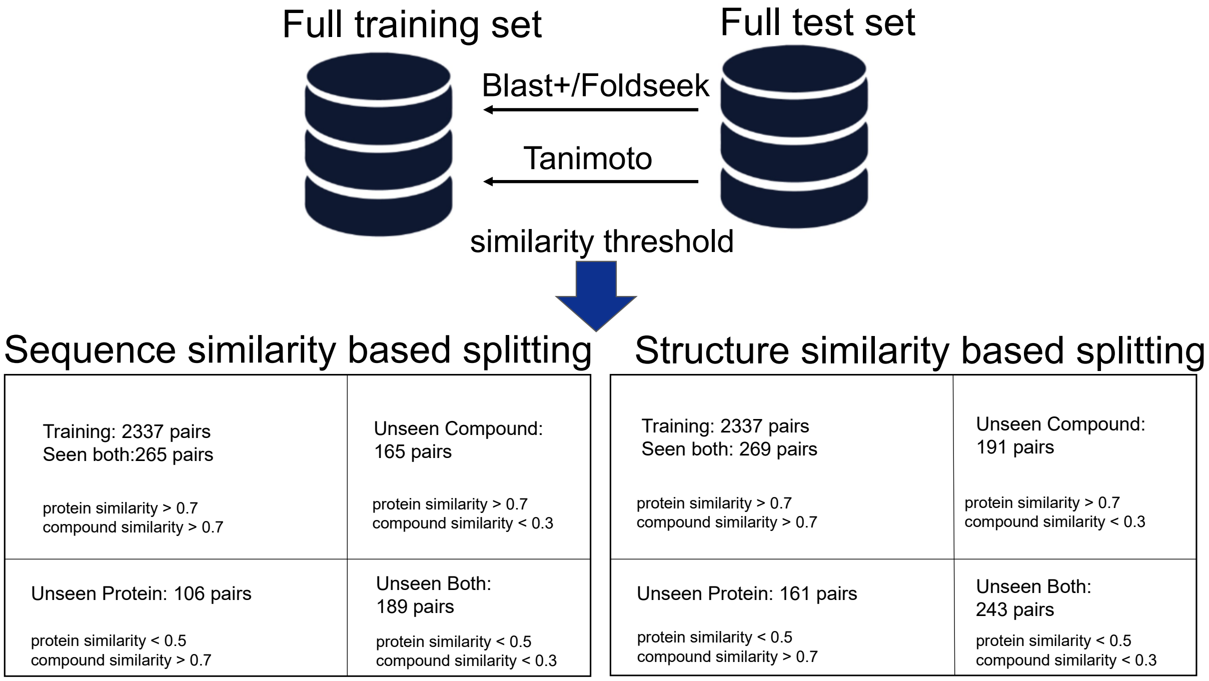

Figure S1. Splitting process based on sequences and structure similarity. The full test set is compared to the training set using BLAST+ for sequence similarity and Foldseek for structure similarity

(A)

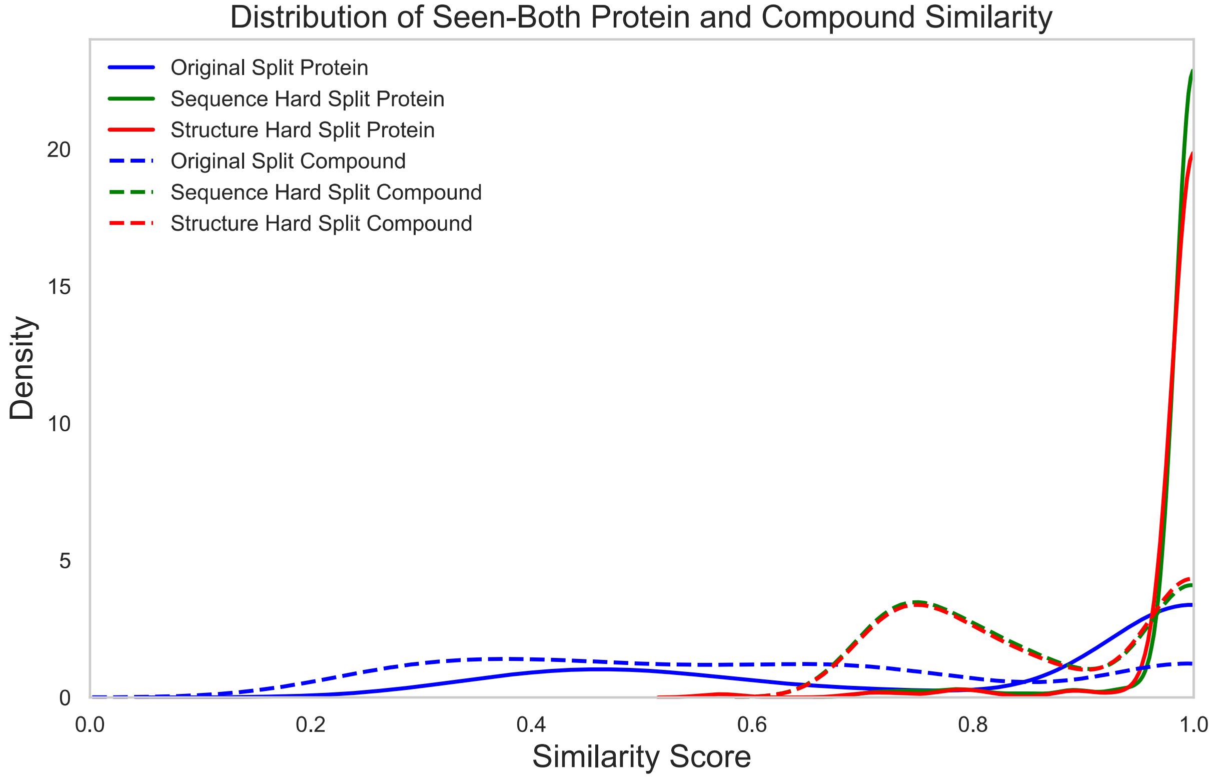
Distribution of Seen-Both Protein and Compound Similarity
Original Split Protein
Sequence Hard Split Protein
Structure Hard Split Protein
Original Split Compound
Sequence Hard Split Compound
Structure Hard Split Compound
Density
0
5
10
15
20
0.0
0.2
0.4
0.6
0.8
1.0
Similarity Score

(B)

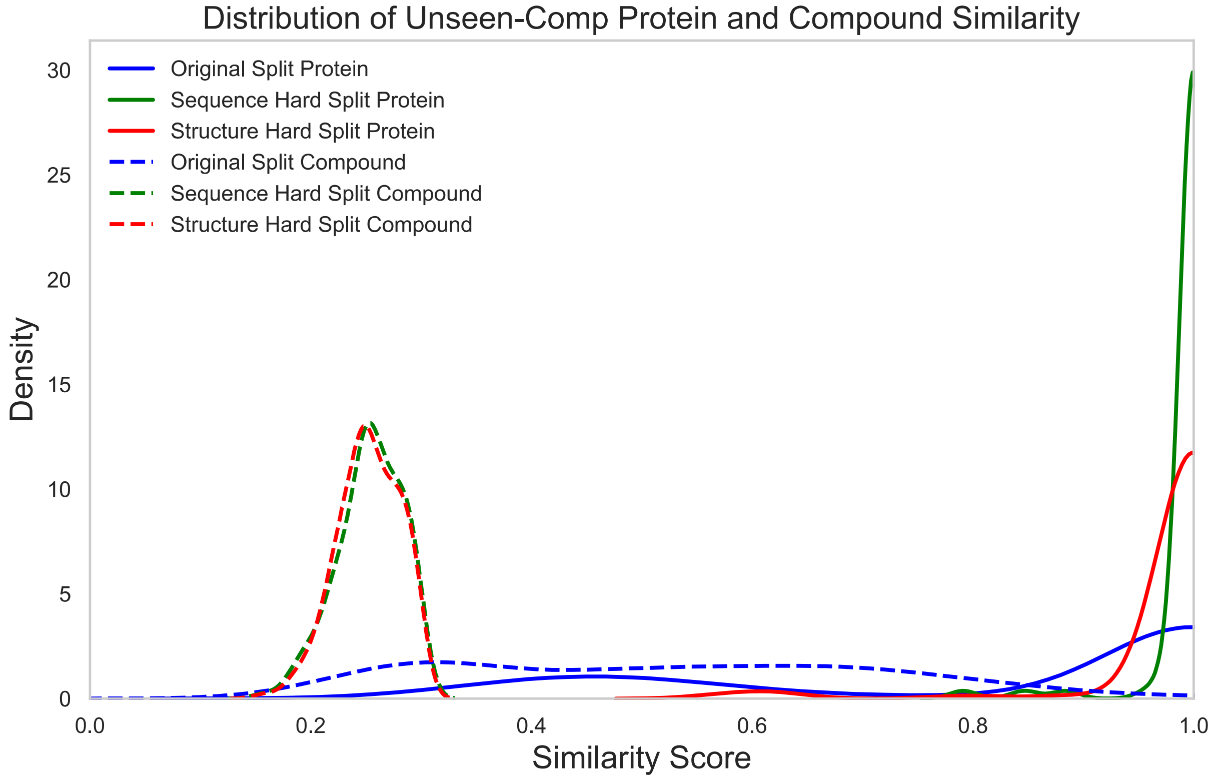
Distribution of Unseen-Comp Protein and Compound Similarity
Original Split Protein
Sequence Hard Split Protein
Structure Hard Split Protein
Original Split Compound
Sequence Hard Split Compound
Structure Hard Split Compound
Density
0
5
10
15
20
25
30
0.0
0.2
0.4
0.6
0.8
1.0
Similarity Score

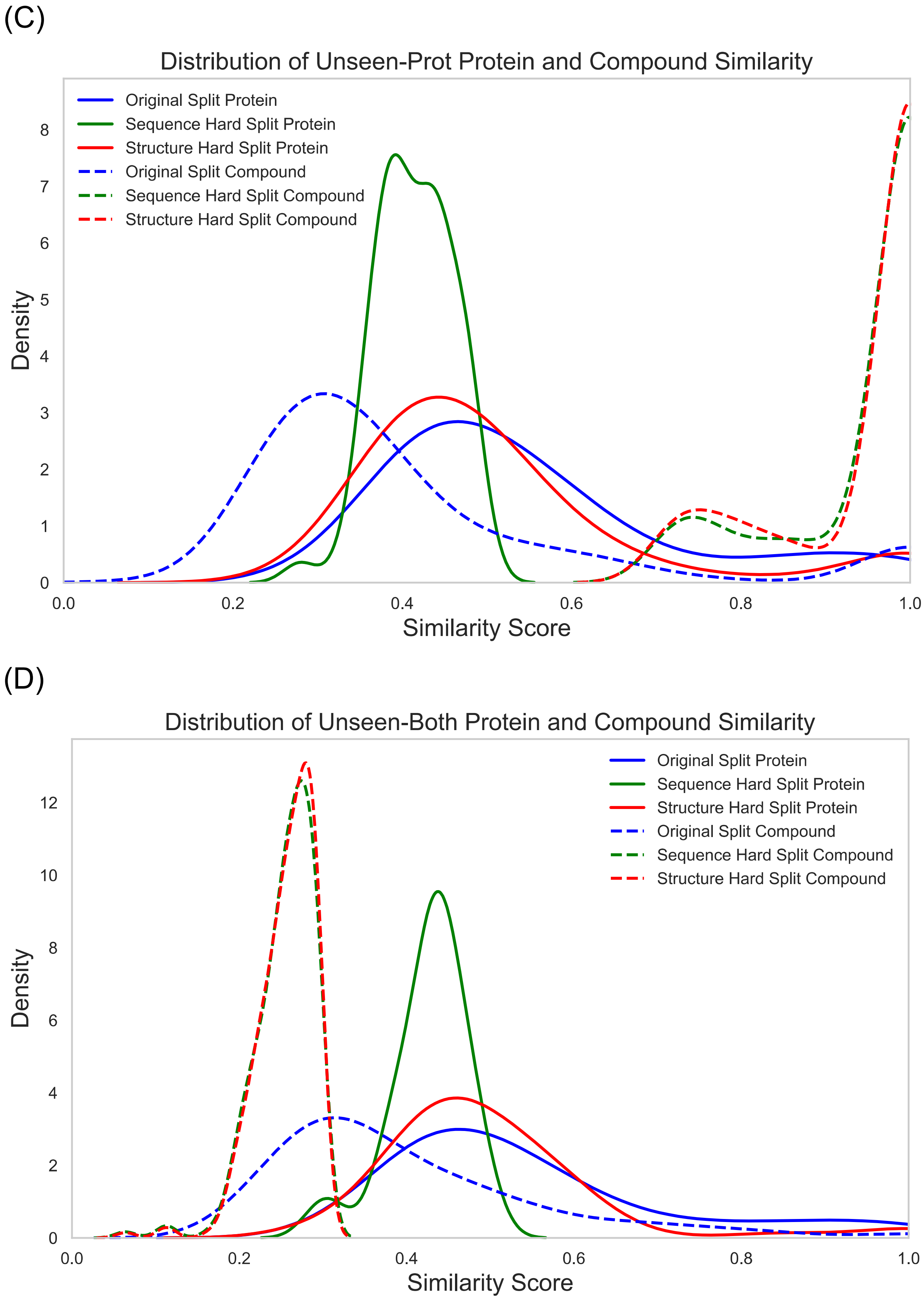
(C)
Distribution of Unseen-Prot Protein and Compound Similarity
Original Split Protein
Sequence Hard Split Protein
Structure Hard Split Protein
Original Split Compound
Sequence Hard Split Compound
Structure Hard Split Compound
Density
0
1
2
3
4
5
6
7
8
0.0
0.2
0.4
0.6
0.8
1.0
Similarity Score
(D)
Distribution of Unseen-Both Protein and Compound Similarity
Original Split Protein
Sequence Hard Split Protein
Structure Hard Split Protein
Original Split Compound
Sequence Hard Split Compound
Structure Hard Split Compound
Density
0
2
4
6
8
10
12
0.0
0.2
0.4
0.6
0.8
1.0
Similarity Score

Figure S2. Distribution of protein and compound similarities across *Original, Sequence Hard and Structure Hard* splits for (A) Seen-Both, (B) Unseen-Comp, (C) Unseen-Prot, and (D) Unseen-Both settings. Protein similarity is shown as solid lines and is defined by blast alignment scores, while compound similarity is shown as dashed lines and is defined by Tanimoto similarity. Each plot compares the similarity scores between training and test sets, highlighting differences in protein and compound similarity for each split type.

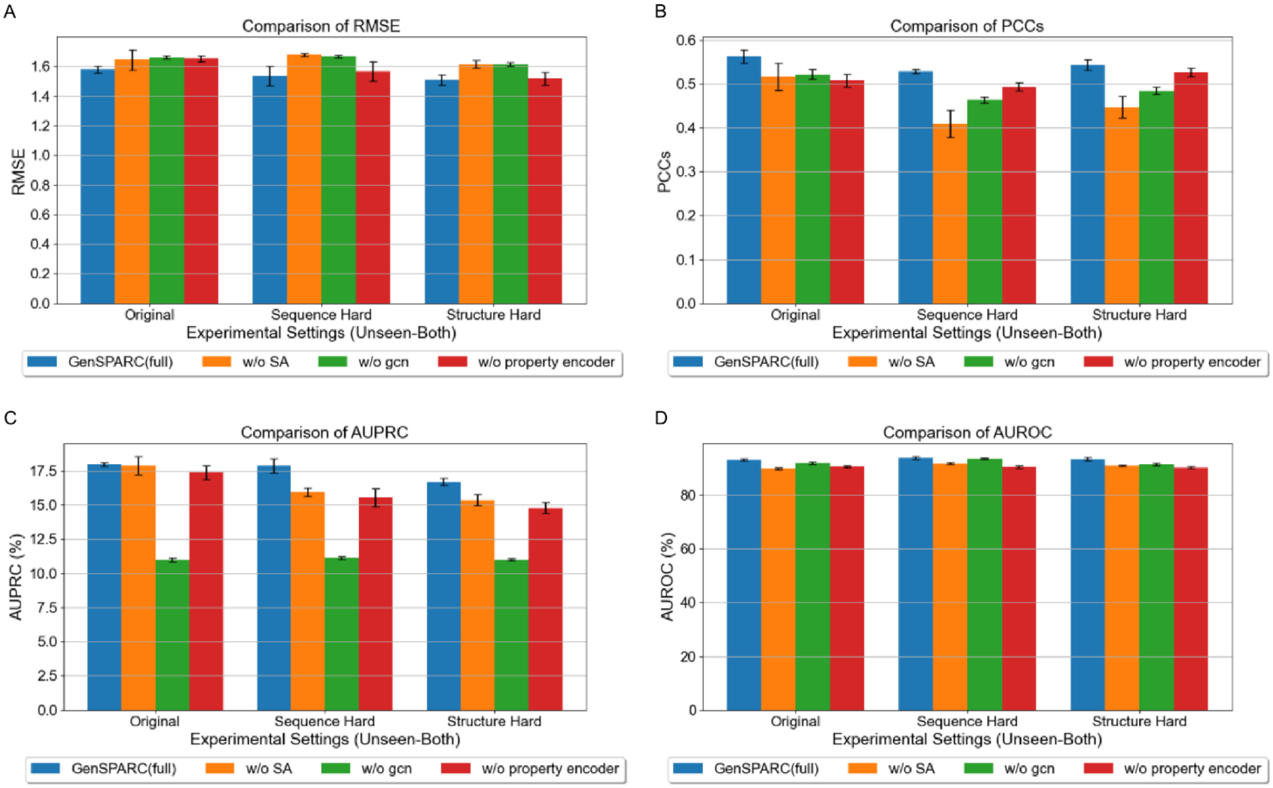

Figure S3. Impact of structure-awareness, GCN, and property encoder on model performance. (A) RMSE, (B) PCC, (C) AUPRC, and (D) AUROC metrics were evaluated for the full GenSPARC model and three alternative configurations: without structure-awareness (w/o SA), without GCN, and without property encoder. Results are shown across the original, sequence-hard, and structure-hard datasets. Data are presented as the mean ± SD over three independent experiments with different random seeds.

1 Methods

1.1 Data processing

1.1.1 Protein sequence

To obtain a structure-aware (SA) sequence for a protein, we first retrieve its Genprot ID. With this ID, we download the predicted structure file from AlphaFold 2[1]. Next, we use Foldseek to process the structure file and generate the SA sequence, where each amino acid residue is followed by a symbol from Foldseek's 3D interaction (3Di) alphabet representing structural information[2]. If a protein does not have a predicted structure, we are unable to create its SA sequence. In such cases, we use the "#" symbol to represent unknown structural information, which in the original Saprot is used to mask the 3Di alphabet.

1.1.2 Molecular Graph Construction from SMILES Strings

In this section, we describe the process of transforming SMILES strings into feature matrices and adjacency matrices for use in the molecular graph encoder. The molecular data, provided as SMILES strings, are first converted into molecular objects using RDKit. Each atom node in a molecule is represented by a rich set of features that capture its chemical properties. These atomic features include:

1. **Aromaticity**: A binary indicator specifying whether the atom is part of an aromatic ring.
2. **Gasteiger Partial Charges**: The partial charges computed using the Gasteiger method to reflect electron distribution.
3. **Formal Charges**: The formal charge of the atom.
4. **Atomic Symbols (One-Hot Encoded)**: A one-hot vector representing the atom type (e.g., C, O, N, etc.).
5. **Hydrogen Bond Donor/Acceptor Properties**: Binary features indicating if the atom can act as a hydrogen bond donor or acceptor.
6. Halogen/Donor Status: Predefined mapping indicating halogen donor properties.
7. **Atomic Degree**: A one-hot vector encoding the number of bonded neighbors (degree ≤ 5). For atoms with degrees greater than 5, a uniform distribution is used.
8. **Total Number of Attached Hydrogens**: A one-hot vector encoding the total hydrogens bonded to the atom.
9. **Implicit Valence**: A one-hot vector representing the implicit valence of the atom.
10. **Number of Radical Electrons**: The number of unpaired electrons associated with the atom.
11. **Hybridization State**: A one-hot vector encoding the hybridization type (e.g., sp, sp2, sp3).
12. **Sanitized Features**: Binary indicators for specific roles, such as single atom donor or acceptor.

For molecules with fewer atoms than the maximum defined size (MAX_size), zero-padding is applied to ensure that all feature matrices have uniform input dimensions required for the model. Each molecule's connectivity is represented by an adjacency matrix, which captures the bonds between atoms and serves as the basis for edge features. The adjacency matrix is padded to a fixed size of MAX_size × MAX_size for consistency. Additionally, a self-loop is added to the adjacency matrix by summing it with an identity matrix, ensuring that each atom includes its own features in its local representation.

The processed feature matrices and adjacency matrices are stored as .npy files for efficient access during training and evaluation. This preprocessing pipeline ensures that the molecular structures are transformed into a consistent, feature-rich format suitable for input into the model.

1.1.3 Data split - *Sequence Hard* and *Structure Hard*

In our study, we created two additional test sets, *Sequence Hard* and *Structure Hard*, to evaluate the generalization of our model under more challenging conditions. Each set was split based on specific thresholds for protein similarity and compound Tanimoto similarity.

Sequence Hard Split

In the *Sequence Hard* split, protein sequence similarity was measured using the BLAST+ [3] software, specifically version "ncbi-blast-2.16.0+-aarch64-macosx.tar.gz", which was downloaded on September 5, 2024. We used this tool to calculate the percentage of identical positions (pident) between protein sequences.

-Seen-Both: Pairs with protein similarity greater than 0.7 and Tanimoto similarity of the compound greater than 0.7. There are 265 pairs in this category.

- Unseen-Comp: Pairs where protein similarity is greater than 0.7 but Tanimoto similarity is less than 0.3. This split resulted in 169 pairs, representing 13.6% of the original Unseen-Comp set.
- Unseen-Prot: Pairs where protein similarity is less than 0.5 and Tanimoto similarity is greater than 0.7. This yielded 106 pairs, covering 6.79% of the original Unseen-Prot set.
- Unseen-Both: Pairs with protein similarity less than 0.5 and Tanimoto similarity less than 0.3. There are 189 pairs in this category, representing 13.4% of the original Unseen-Both set.

Structure Hard Split

In the *Structure Hard* split, protein structure similarity was calculated using Foldseek[2], which aligns protein structures and generates a fraction of identical matches (fident). This method allowed us to cluster the proteins based on structural similarity rather than sequence similarity.

- Seen-Both: Pairs where protein structure similarity (fident) is greater than 0.7 and Tanimoto similarity greater than 0.7. This resulted in 269 pairs, covering 26.7% of the original Seen-Both set.
- Unseen-Comp: Pairs where protein fident is greater than 0.7 and Tanimoto similarity is less than 0.3. We obtained 191 pairs in this category, representing 10.4% of the original set.
- Unseen-Prot: Pairs where protein fident is less than 0.5 and Tanimoto similarity is greater than 0.7, yielding 161 pairs, representing 4.95% of the original Unseen-Prot set.
- Unseen-Both: Pairs where protein fident is less than 0.5 and Tanimoto similarity is less than 0.3. This split resulted in 243 pairs, covering 19.3% of the original Unseen-Both set.

By employing both sequence-based and structure-based splitting methods, we were able to assess how well the model generalizes across various levels of similarity between compounds and proteins, ensuring that the model performs robustly across a wide range of challenging cases.

1.2 Protein Encoder

In our study, we utilize Saprot_650M_AF2 from huggingface as the Pretrained Protein Encoder. SaProt[4] is a Transformer-based model designed to integrate protein sequence and 3D structural information into a unified embedding space. The model employs a dual-token approach, with separate tokenization for amino acid residues and 3D structural conformations. The residue sequence is processed as a series of tokens representing the protein's primary structure, while the 3D structure is encoded using Foldseek into structural tokens. Both token types are embedded

using self-attention mechanisms, producing unimodal feature embeddings that represent both the sequence and structural aspects of the protein.

SaProt is pre-trained on a large dataset comprising 40 million protein sequences and their corresponding 3D structures. The pre-training objectives include masked language modeling (MLM) to predict masked tokens in the sequence, leveraging both structural and residue context. The model uses structure-aware tokens to enhance its ability to capture the relationships between protein sequences and their 3D conformations. SaProt is specifically designed to excel in both protein-level and residue-level tasks, with applications ranging from mutational effect prediction to protein-protein interaction modeling.

1.3 Molecular Graph Encoder

The molecular graph encoder is a graph neural network designed to process graph-structured data by iteratively refining node features while incorporating graph topology. The model consists of three graph propagation layers, each combining a linear transformation, adjacency-based message passing, and non-linear activation. The input features, with a fixed dimension of 43, are first processed by a fully connected linear layer, which maps them into a hidden space of higher dimension, where equals to 512. This transformation is followed by a graph-based message passing operation, implemented using an adjacency tensor that captures the graph structure. For each node, this operation aggregates features from its neighbors based on the adjacency tensor. A ReLU activation function is applied afterward to introduce non-linearity, enabling the model to learn complex feature interactions.

This process is repeated for three consecutive layers, where the updated features from one layer serve as the input to the next. At the final stage, a fully connected output layer maps the features into the same hidden dimension, producing the refined node embeddings. The use of three propagation layers allows the model to progressively capture both local and higher-order graph information. The design ensures that the model is adaptable to various graph-related tasks, with hidden dimension controlling the model's capacity and expressiveness. By leveraging adjacency-aware computations, the model effectively integrates structural and feature information to generate meaningful representations for downstream applications.

1.4 Molecular Property Encoder

The Structure-Property Multi-Modal Foundation Model (SPMM)[5] is a Transformer-based model designed to integrate molecular structure and property information into a shared embedding space. The model employs a dual-stream architecture with separate encoders for SMILES representations and molecular properties. The SMILES encoder processes the molecular structure as a sequence of tokens, while the property vector (PV) encoder handles 53 predefined molecular properties. Both encoders use self-attention mechanisms to generate unimodal feature embeddings, which are then fused through a cross-attention mechanism in a fusion encoder to capture complex structure-property relationships.

SPMM is pre-trained on a large dataset of 50 million molecular SMILES strings from PubChem, with corresponding 53 molecular properties computed using RDKit. The pre-training objectives include contrastive learning to align SMILES and property embeddings, next-word prediction (NWP) for SMILES generation, next-property prediction (NPP) to predict the next property in the sequence, and SMILES-PV matching to ensure correct pairing between structures and properties. The model also employs a momentum teacher to provide pseudo-labels and

enhance learning. In our study, we use only the property vector encoder as the Property Encoder (PE). The 53 molecular properties used are listed in Table S1.

1.5 Molecular Fusion Module

We propose a molecular fusion module to effectively integrate compound and property embeddings. In this module, a single layer consists of one cross-attention operation followed by one self-attention operation. The cross-attention mechanism fuses information from the property embedding into the compound embedding, where the property embedding acts as the query and the compound embedding serves as the key and value. This step enables the model to capture the interactions between the compound and property features. Subsequently, a self-attention operation is applied to the compound embedding, refining it by modeling internal dependencies within the compound features.

The module is designed with flexibility, allowing for an optional masking mechanism that restricts the attention to specific dimensions of the property embedding (e.g., the first 53 features). This ensures that only relevant features are attended to. The fusion module also supports two configurations: shared weights, where the same attention layer is reused across all layers, and unique weights, where each layer is assigned a distinct attention layer.

In our experiments, the module utilizes a single layer of cross-attention and self-attention with shared weights, a hidden dimension of 512, an attention dropout rate of 0.1, and 4 attention heads. This lightweight yet effective design provides a strong capability to capture the interactions between compounds and properties while maintaining computational efficiency.

1.6 Multimodal Attention Network (MAN)

After obtaining the protein and compound embeddings, they are fed into the Multimodal Attention Network (MAN) to capture complex interactions between proteins and compounds. The MAN integrates cross-attention and self-attention mechanisms within a single layer to model both inter-entity interactions and intra-entity dependencies. This process refines the embeddings, enhancing their ability to represent protein-compound relationships effectively.

The network begins by grouping the protein and compound embeddings into smaller subgroups, based on a predefined group size. This grouping reduces computational complexity while preserving essential information. In the **cross-attention** step, protein embeddings act as queries and compound embeddings serve as keys and values, and vice versa. Scaled dot-product attention is used to compute interaction scores, and a masking mechanism ensures that only valid tokens are attended to. The outputs of cross-attention capture how proteins and compounds influence each other, integrating information across both entities.

Following cross-attention, **self-attention** is applied independently to the protein and compound embeddings to capture internal dependencies within each representation. The refined protein embedding is then computed as the mean of its outputs from both self-attention and cross-attention with the compound embedding. Similarly, the compound embedding is updated as the mean of its outputs from both self-attention and cross-attention with the protein embedding. This averaging process ensures that both local and cross-modal information is effectively combined for further refinement.

Residual connections are added after both the attention operations to preserve the original information, and layer normalization is applied to stabilize training. The combined embeddings are further processed by a **feed-forward network (FFN)**, consisting of two fully connected layers with a ReLU activation in between, followed by another layer normalization step. This step allows the network to capture higher-level feature interactions.

In the final layer, the MAN aggregates the refined embeddings by computing the mean of all tokens for both proteins and compounds. The aggregated protein and compound embeddings are concatenated to form the final joint embedding, representing the integrated protein-compound interaction.

In our experiments, the MAN was configured with a hidden dimension of 512, 4 attention heads, and a group size of 1. The feed-forward network expanded the hidden dimension by a factor of 4. This design ensures that the MAN effectively captures both local and global interactions, providing a robust and flexible framework for downstream tasks such as binding affinity prediction.

1.7 Evaluation

We conducted four main types of evaluations for protein-compound interactions: (1) contact map prediction, (2) protein-compound binding affinity, (3) zero-shot virtual screening on DUD-E [6], and (4) cross-validation virtual screening on DUD-E.

1.7.1. Contact Map Prediction: The ground truth contact map is a 2D binary matrix where the rows represent protein residues and the columns represent compound atoms. A value of 1 indicates an interaction, and 0 indicates no interaction. Our model predicts a similar 2D matrix and we evaluate its performance by calculating the Area Under the Precision-Recall Curve (AUPRC) and the Area Under the Receiver Operating Characteristic Curve (AUROC) on the original Karimi [7] dataset, as well as the *Sequence Hard* and *Structure Hard* split datasets.

1.7.2. Protein-Compound Binding Affinity: This is a traditional regression task that predicts the binding strength between a protein and a compound. We report the Root Mean Square Error (RMSE) and Pearson Correlation Coefficients (PCCs) on the original Karimi dataset, *Sequence Hard*, and *Structure Hard* split datasets, following previous works [8,9]. Additionally, we report the Mean Squared Error (MSE) and Concordance Index (CIndex) on the Davis [10], KIBA [11], and Metz [12] datasets as described in [9].

1.7.3. Zero-shot Virtual Screening: In this binary classification task, we predict whether a compound can bind to a protein (1 for active compounds, 0 for decoys). Although AUROC is commonly used for classification, it has been criticized for being less effective in virtual screening due to its focus on false positive rates (FPR) across the entire range (0 to 1). Since virtual screening aims to select a small fraction of active molecules from a large pool, we also use metrics better suited for this scenario: Boltzmann-enhanced discrimination of ROC (BEDROC) and Enrichment Factor (EF) following [13].

1.7.4. Cross-validation Virtual Screening: Similar to zero-shot virtual screening, we evaluate the model using AUROC and ROC Enrichment (RE).

We provide formal definitions and detailed explanations for the key virtual screening metrics: BEDROC, Enrichment Factor (EF), and ROC Enrichment (RE), as shown in the figure.

BEDROC(Boltzmann-enhanced discrimination of ROC): BEDROC incorporates exponential weights to emphasize early-ranked candidates in virtual screening. In particular, the variant BEDROC_{85} is commonly used, where the top 2% of ranked candidates contribute to 80% of the score. This metric is especially useful in scenarios where prioritizing the top-ranked compounds is critical. The formal definition of BEDROC is given by:

$$\text{BEDROC}_\alpha = \frac{\sum_{i=1}^{NTB_\text{t}} e^{-\alpha r_i/N}}{R_\alpha\left(\frac{1-e^{-\alpha}}{e^{\alpha/N}-1}\right)} \times \left(\frac{R_\alpha \sinh(\alpha/2)}{\cosh(\alpha/2) - \cosh(\alpha/2 - \alpha R_\alpha)} + \frac{1}{1-e^{\alpha(1-R_\alpha)}}\right)$$

Enrichment Factor (EF): This metric measures how enriched the top-ranked molecules are for true binders compared to the total number of binders in the pool. EF is calculated as:

$$EF_{\alpha} = \frac{NTB_{\alpha}}{NTB_{t} \times \alpha}$$

where NTB_{α} is the number of true binders in the top α%, and NTB_{t} is the total number of true binders in the dataset.

ROC Enrichment (RE): RE compares the true positive rate to the false positive rate (FPR) at a specific threshold, providing a measure of how well the model performs at identifying active compounds while minimizing false positives. The formula is:

$$RE(x\%) = \frac{TP \times n}{P \times FP_{x\%}}$$

where TP is the number of correctly identified active compounds, P is the total number of active compounds, and $FP_{x\%}$ is the number of false positives predicted at a given FPR threshold, such as 0.5% or 1%

2 Supplementary Tables

Methods	Davis		KIBA		Metz	
	MSE↓	CIndex↑	MSE↓	CIndex↑	MSE↓	CIndex↑
DeepConvDTI	0.294±0.038	**0.875±0.020**	0.397±0.087	**0.789±0.038**	0.337±0.075	0.786±0.020
GraphDTA(GINs)	0.895±0.173	0.628±0.045	1.005±0.160	0.574±0.018	0.953±0.202	0.575±0.031
HyperattentionDTI	0.827±0.263	0.630±0.090	2.432±0.470	0.543±0.016	0.776±0.202	0.541±0.016
TransformerCPI	0.758±0.184	0.655±0.071	0.983±0.170	0.560±0.019	0.723±0.181	0.582±0.040
PeceiverCPI	0.879±0.181	0.629±0.046	1.011±0.151	0.576±0.022	0.967±0.213	0.573±0.032
Cross-Interaction	0.743±0.243	0.597±0.007	0.752±0.090	0.549±0.035	0.646±0.121	0.559±0.035
PSC-CPI	0.732±0.229	0.568±0.044	0.788±0.122	0.542±0.028	0.627±0.096	0.528±0.024
GraphBAN	**0.291±0.068**	0.839±0.066	**0.354±0.087**	0.768±0.043	**0.269±0.115**	**0.799±0.042**
GenSPARC	0.728±0.230	0.592±0.030	0.754±0.047	0.556±0.034	0.611±0.089	0.578±0.043

Table S1. Performance Comparison of GenSPARC with State-of-the-Art Baselines for CPI Strength Prediction under the Seen-Both condition

Performance comparison of GenSPARC with other state-of-the-art baselines for CPI strength prediction on three public datasets under the Seen-Both sequence-hard setting. The best and second metrics are marked with bold and underline, respectively. Data are presented as the mean ± SD of fivefold cross-validation.

Methods	Davis		KIBA		Metz	
	MSE↓	CIndex↑	MSE↓	CIndex↑	MSE↓	CIndex↑
DeepConvDTI	3.938±1.498	0.621±0.018	1.109±0.368	0.651±0.034	3.058±0.684	0.576±0.033
GraphDTA(GINs)	1.229±0.622	0.560±0.035	0.991±0.177	0.552±0.013	0.751±0.274	0.593±0.027
HyperattentionDTI	1.122±0.711	0.525±0.044	2.330±0.318	0.547±0.008	0.708±0.308	0.538±0.023
TransformerCPI	0.941±0.505	0.557±0.031	0.970±0.082	0.569±0.014	0.636±0.126	0.608±0.026
PeceiverCPI	1.416±0.758	0.555±0.028	0.977±0.136	0.555±0.010	0.847±0.393	0.552±0.019
Cross-Interaction	0.923±0.456	0.548±0.027	0.695±0.068	0.531±0.033	0.579±0.085	0.545±0.015
PSC-CPI	0.918±0.459	0.551±0.021	0.691±0.091	0.544±0.027	0.575±0.096	0.540±0.008
GraphBAN	**0.863±0.368**	**0.623±0.025**	**0.511±0.028**	**0.686±0.023**	**0.548±0.076**	**0.651±0.025**
GenSPARC	0.868±0.390	0.527±0.006	0.682±0.026	0.553±0.034	0.572±0.078	0.554±0.024

Table S2. Performance Comparison of GenSPARC with State-of-the-Art Baselines for CPI Strength Prediction under the Unseen-Comp condition

Performance comparison of GenSPARC with other state-of-the-art baselines for CPI strength prediction on three public datasets under the Unseen-Comp sequence-hard setting. The best and second metrics are marked with bold and underline, respectively. Data are presented as the mean ± SD of fivefold cross-validation.

Methods	Davis		KIBA		Metz	
	MSE↓	CIndex↑	MSE↓	CIndex↑	MSE↓	CIndex↑
DeepConvDTI	<u>0.793±0.300</u>	<u>0.679±0.068</u>	0.712±0.253	**0.646±0.018**	<u>0.573±0.059</u>	**0.599±0.013**
GraphDTA(GINs)	1.179±0.570	0.637±0.051	1.022±0.317	0.562±0.015	1.499±0.747	0.504±0.036
HyperattentionDTI	1.261±0.770	0.561±0.020	2.374±0.299	0.517±0.006	0.836±0.376	0.540±0.036
TransformerCPI	0.872±0.332	0.637±0.039	0.766±0.117	0.544±0.016	0.798±0.194	0.500±0.028
PeceiverCPI	1.100±0.428	0.651±0.045	1.017±0.290	0.565±0.014	1.624±0.881	0.494±0.027
Cross-Interaction	0809±0.338	0.550±0.028	<u>0.681±0.031</u>	0.516±0.046	0.623±0.056	0.527±0.030
PSC-CPI	0.827±0.394	0.532±0.027	0.739±0.080	0.517±0.029	0.615±0.042	0.549±0.018
GraphBAN	**0.707±0.259**	**0.695±0.028**	**0.589±0.014**	<u>0.619±0.019</u>	**0.535±0.052**	<u>0.597±0.014</u>
GenSPARC	0.801±0.362	0.542±0.018	0.689±0.029	0.538±0.016	0.599±0.088	0.552±0.047

Table S3. Performance Comparison of GenSPARC with State-of-the-Art Baselines for CPI Strength Prediction under the Unseen-Prot condition

Performance comparison of GenSPARC with other state-of-the-art baselines for CPI strength prediction on three public datasets under the Unseen-Prot sequence-hard setting. The best and second metrics are marked with bold and underline, respectively. Data are presented as the mean ± SD of fivefold cross-validation.

Property Name	RDKit Function Name
BalabanJ	rdkit.Chem.GraphDescriptors.BalabanJ
BertzCT	rdkit.Chem.GraphDescriptors.BertzCT
Chi0	rdkit.Chem.GraphDescriptors.Chi0
Chi0n	rdkit.Chem.GraphDescriptors.Chi0n
Chi0v	rdkit.Chem.GraphDescriptors.Chi0v
Chi1	rdkit.Chem.GraphDescriptors.Chi1
Chi1n	rdkit.Chem.GraphDescriptors.Chi1n
Chi1v	rdkit.Chem.GraphDescriptors.Chi1v
Chi2n	rdkit.Chem.GraphDescriptors.Chi2n
Chi2v	rdkit.Chem.GraphDescriptors.Chi2v
Chi3n	rdkit.Chem.GraphDescriptors.Chi3n
Chi3v	rdkit.Chem.GraphDescriptors.Chi3v
Chi4n	rdkit.Chem.GraphDescriptors.Chi4n
Chi4v	rdkit.Chem.GraphDescriptors.Chi4v
ExactMolWt	rdkit.Chem.Descriptors.ExactMolWt
FpDensityMorgan1	rdkit.Chem.AllChem.GetMorganFingerprintAsBitVect
FpDensityMorgan2	rdkit.Chem.AllChem.GetMorganFingerprintAsBitVect
FpDensityMorgan3	rdkit.Chem.AllChem.GetMorganFingerprintAsBitVect
FractionCSP3	rdkit.Chem.Descriptors.FractionCSP3
HallKierAlpha	rdkit.Chem.Descriptors.HallKierAlpha
HeavyAtomCount	rdkit.Chem.Lipinski.HeavyAtomCount
HeavyAtomMolWt	rdkit.Chem.Descriptors.HeavyAtomMolWt
Kappa1	rdkit.Chem.Descriptors.Kappa1
Kappa2	rdkit.Chem.Descriptors.Kappa2
Kappa3	rdkit.Chem.Descriptors.Kappa3
LabuteASA	rdkit.Chem.rdMolDescriptors.CalcLabuteASA
MaxAbsEStateIndex	rdkit.Chem.Descriptors.MaxAbsEStateIndex
MaxEStateIndex	rdkit.Chem.Descriptors.MaxEStateIndex
MinAbsEStateIndex	rdkit.Chem.Descriptors.MinAbsEStateIndex
MinEStateIndex	rdkit.Chem.Descriptors.MinEStateIndex
MolLogP	rdkit.Chem.Crippen.MolLogP
MolMR	rdkit.Chem.Crippen.MolMR
MolWt	rdkit.Chem.Descriptors.MolWt
NHOHCount	rdkit.Chem.Lipinski.NHOHCount
NOCount	rdkit.Chem.Lipinski.NOCount
NumAliphaticCarbocycles	rdkit.Chem.rdMolDescriptors.CalcNumAliphaticCarbocycles
NumAliphaticHeterocycles	rdkit.Chem.rdMolDescriptors.CalcNumAliphaticHeterocycles
NumAliphaticRings	rdkit.Chem.rdMolDescriptors.CalcNumAliphaticRings
NumAromaticCarbocycles	rdkit.Chem.rdMolDescriptors.CalcNumAromaticCarbocycles
NumAromaticHeterocycles	rdkit.Chem.rdMolDescriptors.CalcNumAromaticHeterocycles
NumAromaticRings	rdkit.Chem.rdMolDescriptors.CalcNumAromaticRings
NumHAcceptors	rdkit.Chem.Lipinski.NumHAcceptors
NumHDonors	rdkit.Chem.Lipinski.NumHDonors
NumHeteroatoms	rdkit.Chem.rdMolDescriptors.CalcNumHeteroatoms

NumRadicalElectrons	rdkit.Chem.rdMolDescriptors.CalcNumRadicalElectrons
NumRotatableBonds	rdkit.Chem.Lipinski.NumRotatableBonds
NumSaturatedCarbocycles	rdkit.Chem.rdMolDescriptors.CalcNumSaturatedCarbocycles
NumSaturatedHeterocycles	rdkit.Chem.rdMolDescriptors.CalcNumSaturatedHeterocycles
NumSaturatedRings	rdkit.Chem.rdMolDescriptors.CalcNumSaturatedRings
NumValenceElectrons	rdkit.Chem.rdMolDescriptors.CalcNumValenceElectrons
RingCount	rdkit.Chem.rdMolDescriptors.CalcNumRings
TPSA	rdkit.Chem.rdMolDescriptors.CalcTPSA
QED	rdkit.Chem.QED.qed

Table S4. List of 53 Molecular Properties and Corresponding RDKit Functions for Calculation.

Methods	Seen-Both		Unseen-Comp		Unseen-Prot		Unseen-Both	
	AUPRC↑	AUROC↑	AUPRC↑	AUROC↑	AUPRC↑	AUROC↑	AUPRC↑	AUROC↑
Original split								
GenSPARC(full)	**25.44**	90.54	**24.87**	**91.57**	**20.00**	**93.82**	**18.19**	93.24
w/o SA	23.12	85.49	22.14	87.00	19.25	89.46	17.22	89.52
w/o gcn	14.86	**91.13**	13.90	91.30	11.12	93.58	10.83	92.13
w/o property	24.69	88.10	24.64	88.91	19.40	91.54	18.09	91.21
Sequence Hard								
GenSPARC(full)	**33.25**	**91.52**	**12.60**	**91.21**	**25.34**	**92.89**	**18.54**	**94.12**
w/o SA	31.20	86.78	10.77	80.53	23.42	88.46	15.52	91.85
w/o gcn	14.86	91.13	7.91	90.42	15.28	92.04	11.01	94.00
w/o property	32.92	89.35	12.47	87.66	22.81	90.70	16.47	91.08
Structure Hard								
GenSPARC(full)	**32.72**	**91.69**	**11.57**	**90.99**	**23.75**	**91.24**	**16.78**	**93.44**
w/o SA	31.77	87.55	10.73	81.45	21.58	87.24	14.76	91.01
w/o gcn	14.99	91.53	8.03	90.54	13.47	90.12	10.89	91.72
w/o property	31.13	90.01	9.94	87.68	20.68	88.30	15.35	90.83

Table S5. Ablation study results of GenSPARC on various splits for protein-compound interaction contact map prediction. The best and second metrics are marked with bold and underline, respectively.

Methods	Seen-Both		Unseen-Comp		Unseen-Prot		Unseen-Both	
	RMSE↓	PCCs↑	RMSE↓	PCCs↑	RMSE↓	PCCs↑	RMSE↓	PCCs↑
Original split								
GenSPARC(full)	**1.357**	**0.748**	**1.221**	**0.787**	**1.551**	**0.526**	**1.552**	**0.583**
w/o SA	1.405	0.724	1.294	0.749	1.578	0.500	1.607	0.535
w/o gcn	1.480	0.691	1.270	0.765	1.633	0.465	1.655	0.528
w/o property	1.407	0.722	1.291	0.757	1.529	0.525	1.635	0.520
Sequence Hard								
GenSPARC(full)	**1.121**	**0.805**	**1.372**	**0.631**	**1.444**	**0.642**	**1.624**	**0.522**
w/o SA	1.165	0.784	1.395	0.614	1.699	0.487	1.677	0.452
w/o gcn	1.198	0.772	1.400	0.610	1.922	0.374	1.662	0.465
w/o property	1.151	0.795	1.384	0.617	1.565	0.575	1.654	0.492
Structure Hard								
GenSPARC(full)	**1.141**	**0.793**	1.411	0.618	**1.519**	**0.582**	**1.559**	**0.546**
w/o SA	1.190	**0**.768	1.472	0.595	1.599	0.516	1.580	0.479
w/o gcn	1.263	0.734	1.490	0.605	1.821	0.411	1.614	0.477
w/o property	1.198	0.777	1.483	0.609	1.579	0.532	1.577	0.521

Table S6. Ablation study results of GenSPARC on various splits for protein-compound interaction binding affinity prediction. The best and second metrics are marked with bold and underline, respectively.

Feature	Description	Dimension
Aromaticity	Binary indicator of whether the atom is aromatic.	1
Gasteiger Partial Charges	Partial charge reflecting electron distribution around the atom.	1
Formal Charge	Formal charge of the atom.	1
Atomic Symbol (One-Hot Encoded)	One-hot vector representation of the atom type.	13
Hydrogen Bond Donor/Acceptor	Binary indicators for donor and acceptor roles.	2
Halogen/Donor Status	Predefined mapping indicating halogen donor properties.	1
Atomic Degree	One-hot vector of bonded neighbors (degree ≤ 5).	6
Total Number of Hydrogens	One-hot vector encoding the number of hydrogens attached to the atom.	5
Implicit Valence	One-hot vector of the atom's implicit valence.	6
Number of Radical Electrons	Number of unpaired electrons associated with the atom.	1
Hybridization State	One-hot vector of the atom's hybridization type (sp, sp2, sp3, sp3d, sp3d2).	Length of dictionary + 1
Sanitized Features	Binary indicators for specific atomic roles (e.g., donor/acceptor).	2

Table S7. List of Atomic Features Used for Molecular Graph Representation.

References

1 Jumper, J. *et al.* Highly accurate protein structure prediction with AlphaFold. *Nature* **596**, 583-589 (2021).
2 Van Kempen, M. *et al.* Fast and accurate protein structure search with Foldseek. *Nature biotechnology* **42**, 243-246 (2024).
3 Camacho, C. *et al.* BLAST+: architecture and applications. *BMC bioinformatics* **10**, 1-9 (2009).
4 Su, J. *et al.* Saprot: Protein language modeling with structure-aware vocabulary. *bioRxiv*, 2023.2010. 2001.560349 (2023).
5 Chang, J. & Ye, J. C. Bidirectional generation of structure and properties through a single molecular foundation model. *Nature Communications* **15**, 2323 (2024).
6 Mysinger, M. M., Carchia, M., Irwin, J. J. & Shoichet, B. K. Directory of useful decoys, enhanced (DUD-E): better ligands and decoys for better benchmarking. *Journal of medicinal chemistry* **55**, 6582-6594 (2012).
7 Karimi, M., Wu, D., Wang, Z. & Shen, Y. Explainable deep relational networks for predicting compound–protein affinities and contacts. *Journal of chemical information and modeling* **61**, 46-66 (2020).
8 You, Y. & Shen, Y. Cross-modality and self-supervised protein embedding for compound–protein affinity and contact prediction. *Bioinformatics* **38**, ii68-ii74 (2022).
9 Wu, L. *et al.* in *Proceedings of the AAAI Conference on Artificial Intelligence.* 310-319.
10 Davis, M. I. *et al.* Comprehensive analysis of kinase inhibitor selectivity. *Nature biotechnology* **29**, 1046-1051 (2011).
11 Tang, J. *et al.* Making sense of large-scale kinase inhibitor bioactivity data sets: a comparative and integrative analysis. *Journal of Chemical Information and Modeling* **54**, 735-743 (2014).
12 Metz, J. T. *et al.* Navigating the kinome. *Nature chemical biology* **7**, 200-202 (2011).
13 Gao, B. *et al.* Drugclip: Contrasive protein-molecule representation learning for virtual screening. *Advances in Neural Information Processing Systems* **36** (2024).
